# Data on analysis of OCC-1 transcript levels in pluripotent and differentiated states of P19 cells

**DOI:** 10.1016/j.dib.2020.105367

**Published:** 2020-02-29

**Authors:** Zahra Hosseininia, Sara Soltanian, Naser Mahdavi-Shahri, Hesam Dehghani

**Affiliations:** a Division of Biotechnology, Faculty of Veterinary Medicine, Ferdowsi University of Mashhad, Mashhad, Iran; b Stem Cells and Regenerative Medicine Research Group, Research Institute of Biotechnology, Ferdowsi University of Mashhad, Mashhad, Iran; c Department of Biology, Faculty of Sciences, Shahid Bahonar University of Kerman, Kerman, Iran; d Department of Biology, Faculty of Sciences, Ferdowsi University of Mashhad, Mashhad, Iran

**Keywords:** OCC-1, P19, Pluripotency, Differentiation, Retinoic acid

## Abstract

We investigated the expression of OCC-1 at mRNA level during retinoic acid (RA) induced differentiation of mouse embryonic carcinoma P19 pluripotent cancer cells by quantitative real time PCR (qPCR). By employing four-fold serial dilutions of P19 cDNA, standard curves were generated for the reference gene (L37) and the gene of interest (OCC-1). PCR efficiencies for L37 and OCC-1 were calculated. Since the amplification efficiencies of these two genes were unequal, the standard curve method was used for the relative quantification of OCC-1. Data analysis revealed that the expression of OCC-1 was reduced by about 69% after 4-day treatment with RA, when significant down-regulation of key pluripotency factors, including OCT4 and Nanog was observed [1].

**Table:** Specifications Table

Subject	Molecular Biology
Specific subject area	Down-regulation of OCC-1 after RA-induced differentiation in P19 cells
Type of data	Table Graph Figure Text file
How data were acquired	Cell culture, differentiation of P19 cells, RT-PCR, RT-qPCR
Data format	Raw and analyzed data
Parameters for data collection	Differentiation induction of embryonic carcinoma (EC) P19 cells with retinoic acid (RA).
Description of data collection	The expression of OCC-1 was measured in undifferentiated control and RA-induced differentiated P19 cells by RT-qPCR.
Data source location	Laboratory of Biotechnology, Division of Biotechnology, Faculty of Veterinary Medicine, AND Research Institute of Biotechnology, Ferdowsi University of Mashhad, Mashhad, Iran.
Data accessibility	All data are presented in this article

**Table:** 

**Value of the Data** • To our knowledge, these data for the first time report the presence and expression of OCC-1 in P19 cells which are pluripotent cancer cells. These data are useful because detection of pluripotency associated genes can serve as markers for evaluating the malignancy status of tumor cells. • The data will be valuable for researchers who are interested in identifying new isoforms and functions of mouse OCC-1 and the probable role in cancer and pluripotency state. • OCC-1 was reported as a pluripotency-associated gene in human [2] and these data reinforce this relationship in mouse. Thus, these data will stimulate other scientists to reveal the underlying relationship between different isoforms of OCC-1 and pluripotency and probable role of OCC-1 in pluripotency network.

## 1 Data description

**Fig. 1:**
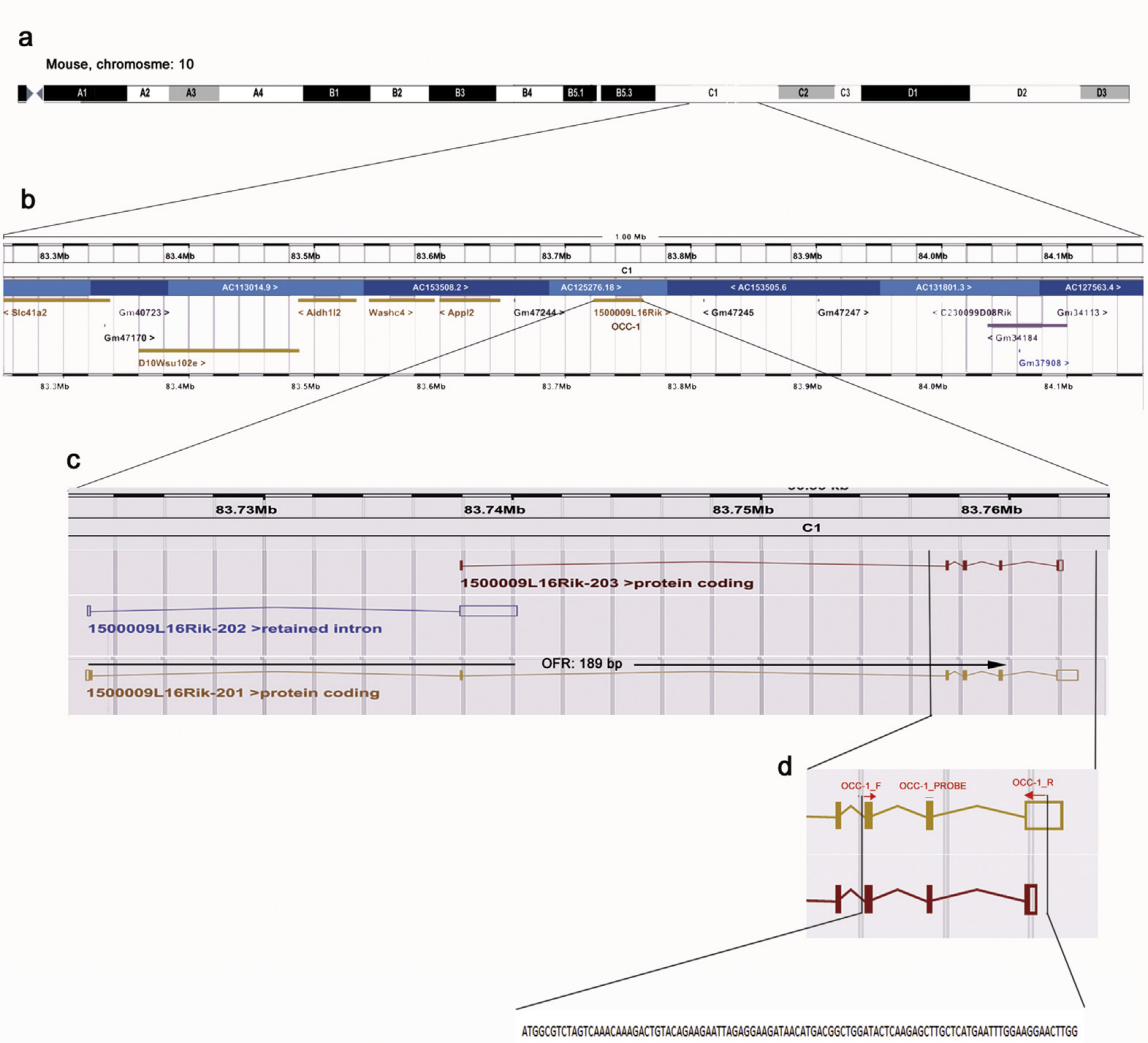
**The schematic representation of the OCC-1 gene on the mouse chromosome 10.** OCC-1 gene and OCC-1 neighboring genes on chromosome 10 (a, b). Among the three predicted isoforms of OCC-1 gene (c), our PCR reaction could amplify a region from two coding isoforms of 1500009L16Rik-201 and 1500009L16Rik-203. The red arrows show the forward and reverse primers (d). The probe-binding site is shown as a red bar (d).

**Fig. 2:**
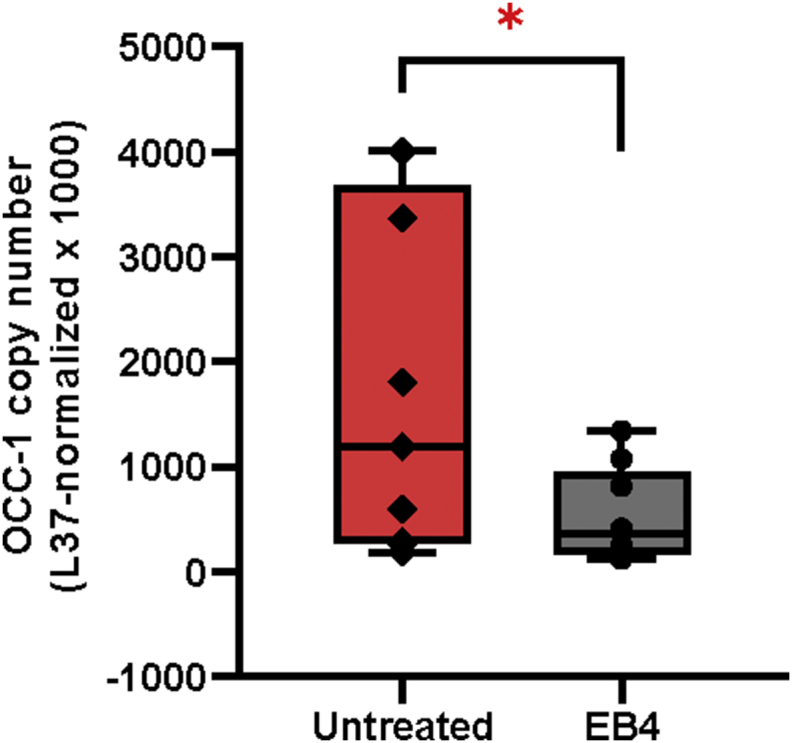
**OCC-1 copy number before and after RA treatment.** Quantitative RT-PCR analysis showed significant down-regulation of OCC-1 copy number after 4 days of RA treatment of P19 cells. The aggregated P19 cells after 4 days of treatment with RA are named EB4. The OCC-1 copy number was normalized by L37 as a reference gene.

**Fig. 3:**
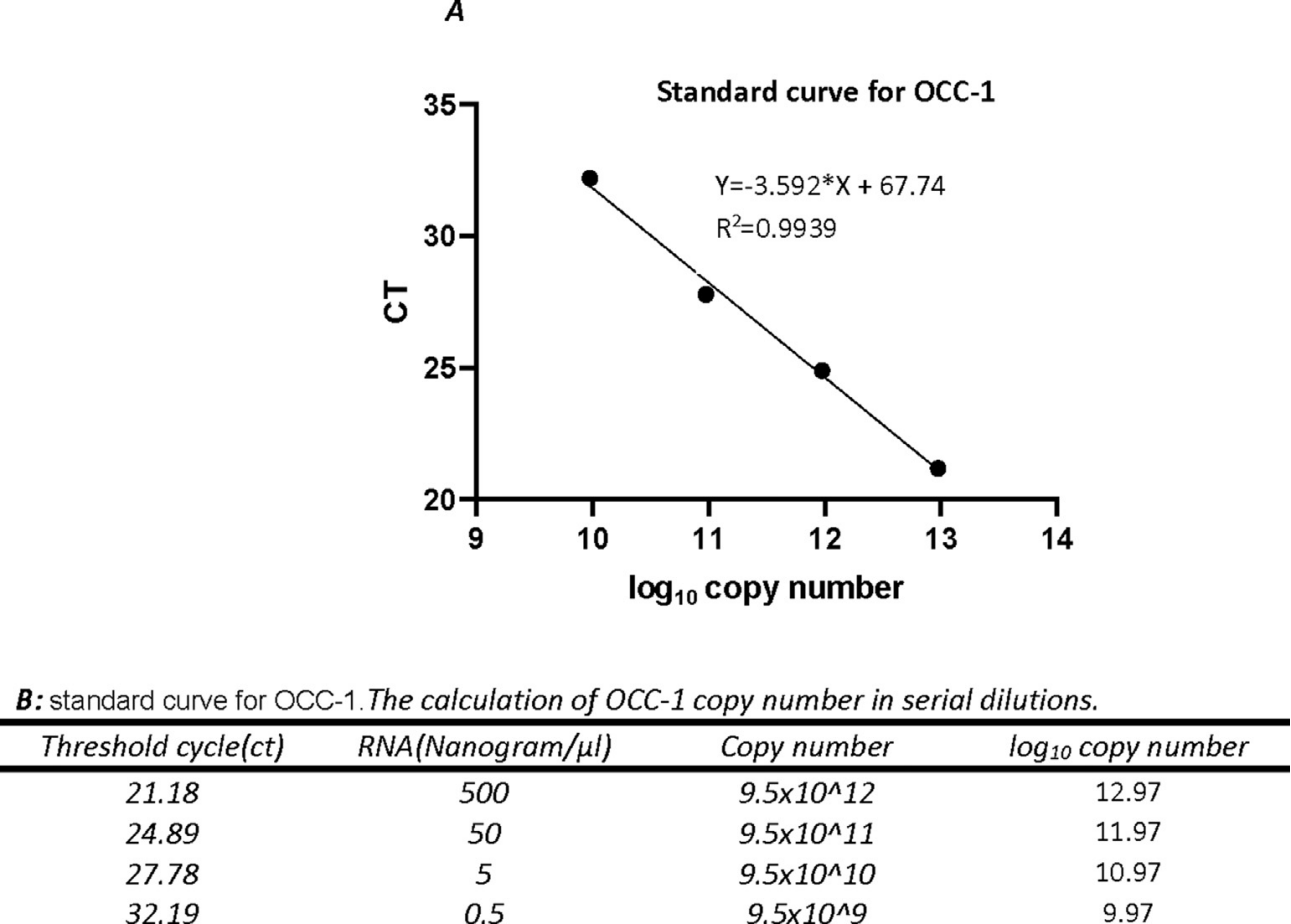
**Standard curve for OCC-1. A)** Standard curve for OCC-1 is drawn by using log copy number (X axes) and CT (Y axes). B) Fourfold serial dilutions of P19 cDNA was used to draw OCC-1 standard curve. Copy number of OCC-1 product in each dilution was calculated according to this estimate that there are 1.9 × 10^12^ mRNA molecules with an average size of 1000 base in 1 μg of total RNA (Table 2).

In mouse, the Ensembl database reports three isoforms including two coding (1500009L16Rik-201 and 1500009L16Rik-203) and one non-coding RNA on the chromosome 10 (1500009L16Rik-202) (Fig. 1). The expression of OCC-1 in EC P19 cells was analyzed by RT-PCR. PCR reaction with OCC-1 specific primers yielded a 100-bp fragment which was verified by Sanger sequencing (Fig. 1, and Table 1). In order to quantify and compare the relative mRNA levels of OCC-1 before and after RA-induced differentiation of P19 cells, three biological replicates for both untreated (control) and RA-treated (test) P19 cells were subjected to RNA extraction and cDNA synthesis. Then, quantitative real-time PCR (RT-qPCR) was performed in triplicates (Fig. 2). Fourfold serial dilutions of P19 cDNA was used to generate standard curves for OCC-1 and L37. Slope of standard curves was used to calculate amplification efficiencyies of L37 and OCC-1 (Fig. 3 and Table 2). Data analysis using standard curve method showed that transcript level of OCC-1 was reduced to approximately 69% in 4 day- RA-treated P19 cells in comparison with undifferentiated P19 cells (Fig. 2 and Table 3).

**Table 1:** Sequences of primers and probes used in the study.

Gene	Primer and probe sequence (5′–3′)	Product length (bp)
Overexpressed in Colon Carcinoma-1 protein (OCC-1) NM_001145198.1	OCC-1_F: ATGGCGTCTAGTCAAACA OCC-1_R: CCAAGTTCCTTCCAAATTCA OCC-1 Probe: FAM-ATCCAGCCGTCATGTTATCTTCCT-TAMRA	100 bp
Ribosomal protein L37 (Rpl37) NM_026069.3	L37_F: GCAGATTCAGACATGGATTC L37_R: GGAAGAAGCGTAGGATCC L37 Probe: HEX-TCATATAACCGAACTCTGAACCGATGT-BHQ1	200 bp

**Table 2:** Estimation of copy number of OCC-1 and L37.

	RNA (Nanogram)	aCopy number of mRNA	Average size (bp)
(Bustin et al., 1999)	1000	1.9∗10ˆ12	1000
OCC-1	1000	1.9∗10ˆ13	100
L37	1000	0.38∗10ˆ12	200

a The copy number of amplicons in 1 μg total RNA for each gene is calculated based on the estimation that there are 1.9 × 10^12^ mRNA molecules with an average size of 1000 base in 1 μg of total RNA (explained in Experimental Design, Materials, and Methods).

**Table 3:** Quantification of OCC-1 transcript changes following RA treatment using the standard curve method of relative quantification.

	Sample^a^	OCC-1 transcript			L37 transcript			bNorma-lized copy number of OCC-1 (x1000)	Average of Norma-lized OCC-1 copy number	Fold change of OCC-1 After treatment
		cycle threshold (ct)	quantity (log copy number)	Anti-log of copy number	cycle thres-hold (ct)	quantity (log copy number)	Anti-log of copy number			
Untreated	P19 1-1	22.50	12.60111421	3.9913E+12	22.86	13.14673291	1.40195E+13	284.69	1745.43	0.3 (69% reduction)
	P19 1-1	22.67	12.55376045	3.57899E+12	22.90	13.13468835	1.3636E+13	262.46		
	P19 1-1	22.68	12.55097493	3.55611E+12	22.43	13.27621198	1.88891E+13	188.26		
	P19 1-2	22.28	12.66239554	4.59616E+12	25.32	12.40599217	2.54678E+12	1804.69		
	P19 1-2	21.00	13.0189415	1.04458E+13	25.21	12.43911472	2.74862E+12	4000.46		
	P19 1-2	20.92	13.04122563	1.09958E+13	25.00	12.50234869	3.17943E+12	3370.81		
	P19 1-3	20.96	13.03008357	1.07173E+13	25.25	12.42707016	2.67344E+12	4008.79		
	P19 1-3	22.07	12.72089136	5.25886E+12	23.53	12.94498645	8.81021E+12	596.90		
	P19 1-3	21.50	12.87966574	7.57994E+12	24.00	12.80346281	6.36008E+12	1191.79		
Treated (B4)	P19 4-1	23.36	12.36155989	2.29911E+12	23.28	13.02026498	1.04777E+13	219.42	524.08	
	P19 4-1	22.80	12.51754875	3.29267E+12	23.50	12.95401987	8.99539E+12	366.04		
	P19 4-1	23.00	12.46183844	2.89627E+12	23.66	12.90584161	8.05085E+12	408.98		
	P19 4-2	21.79	12.79888579	6.29341E+12	22.10	13.37557964	2.37454E+13	121.97		
	P19 4-2	22.33	12.64846797	4.45111E+12	21.49	13.55925926	3.62459E+13	122.80		
	P19 4-2	22.00	12.74038997	5.50035E+12	22.21	13.34245709	2.20017E+13	249.99		
	P19 4-3	21.65	12.83788301	6.88467E+12	24.31	12.71128018	5.14375E+12	1338.45		
	P19 4-3	22.29	12.65961003	4.56678E+12	24.18	12.74850653	5.60411E+12	814.89		
	P19 4-3	22.10	12.71253482	5.15864E+12	24.41	12.68143972	4.80219E+12	1074.22		

a Samples identified as P19 1-1, 1–2 and 1–3 are three replicates of undifferentiated P19 control cells, and samples identified as P19 4–1, 4–2 and 4–3 are three replicates of 4-day RA-treated differentiated aggregates of P19 (EB4) cells.

b Normalized OCC-1 copy numbers were acquired by dividing OCC-1 transcript copy numbers to L37 transcript copy numbers.

## 2 Experimental design, materials, and methods

### 2.1 Cell culture and RA-induced differentiation

P19 cells were obtained from Pasteur Institute (Tehran, Iran). They were subjected to differentiation by seeding at a density of 0.2 × 10^6^ in 8-cm diameter bacteriological petri dishes at the presence of α-MEM medium supplemented with 0.5μM RA. After 4 days of RA treatment, differentiated aggregates (EB4) were formed and used for RNA extraction.

### 2.2 Quantitative real-time PCR (RT-qPCR)

Total RNA was isolated using the Total RNA Isolation Kit (DENAzist Asia Co., Mashhad, Iran) according to the manufacture's protocol. The quality and quantity of RNA were evaluated by agarose gel electrophoresis and Nanodrop spectrophotometry. First strand cDNA was generated in the presence of 1mM dNTPs, 0.5 μg Oligo(dT)18 primer, 1x reverse transcription buffer, and 200 U of reverse transcriptase in a total volume of 20 μl. The reaction was incubated for 60 min at 42 °C followed by enzyme inactivation at 72 °C for 10 min.

The primers and hydrolysis probes for RT-qPCR were designed using “Beacon Designer” software (Table 1). qPCR reaction was performed in a total volume of 25 μl which contained 1 μl cDNA, 1x PCR buffer, 0.4 mM dNTPs, 1.5 mM MgCl_2_, 200 nM probe, 200 nM of each primer, and 0.04 U of Taq DNA polymerase in a Rotor Gene Q real time thermocycler (Qiagen, USA). All reaction components were purchased from Genet Bio (Daejeon, South Korea). The thermocycler program for OCC-1 and L37 was an initial step of 94 °C (3 min), followed by 40 cycles of 94 °C (30 s), 58 °C (30 s), and 72 °C (30 s).

In order to calculate the PCR efficiency, standard curves for OCC-1 and L37 were generated by employing fourfold serial dilutions of P19 cDNA. The PCR reactions at each serial dilution were performed in triplicates. It is estimated that there are 1.9 × 10^12^ mRNA molecules with an average size of 1000 base in 1 μg of total RNA [3]. Accordingly, we estimated the copy number data of amplicons in 1 μg total RNA for each gene (Table 2). The standard curves were plotted with the data of Ct values on the “Y” axis and the log_10_ copy number of each transcript on the “X” axis (Fig. 3). The slope (S) of the lines were used to calculate PCR efficiencies. The correlation (R^2^) was 0.99 for OCC-1 and L37 and the amplification efficiencies for OCC-1 and L37 were 89.8% and 100%, respectively. Since the amplification efficiencies of the reference gene (L37) and the gene of interest (OCC-1) were unequal, the standard curve method for relative quantification was used [4].

Three biological replicates were considered for both untreated (control) and RA-treated (test) P19 cells. The value data of Ct for each triplicate was extrapolated onto the standard curve to calculate the copy number data of the transcripts in each group (Table 3). Here, L37 was used as the internal reference gene because its transcription did not change after RA-treatment [1]. To determine changes in the transcript levels of the target gene (OCC-1) after RA treatment, the data were analyzed by the following equations [5]:

$$\text{ Normalized target }\left(\text{test sample}\right)\;=\;\frac{\text{Copy number of OCC}-1}{\text{Copy number of L}37}$$

$$\text{Normalized target }\left(\text{control sample}\right)\;=\;\frac{\text{copy number of OCC}-1}{\text{copy number of L}37}$$

$$\text{Fold difference in the level of target transcript}\;=\;\frac{\text{Normalized target }\left(\text{test sample}\right)}{\text{Normalized target }\left(\text{control sample}\right)}$$

As the data in Table 3 indicated, a 69% reduction for OCC-1 was acquired.

### 2.3 Statistical analysis

A minimum of three replicate experiments were performed for all data. Analysis using Mann Whitney test demonstrated a statistical difference between the control and test groups at a *p*-value of less than 0.05.
